# Proteomic Analysis of Vero Cells Infected with Pseudorabies Virus

**DOI:** 10.3390/v14040755

**Published:** 2022-04-04

**Authors:** Xintan Yang, Shengkui Xu, Dengjin Chen, Ruijiao Jiang, Haoran Kang, Xinna Ge, Lei Zhou, Jun Han, Yongning Zhang, Xin Guo, Hanchun Yang

**Affiliations:** Key Laboratory of Animal Epidemiology of the Ministry of Agriculture, College of Veterinary Medicine, China Agricultural University, Beijing 100193, China; s20193050751@cau.edu.cn (X.Y.); skxu0721@163.com (S.X.); chendengjin-cau@foxmail.com (D.C.); wn2jjj@163.com (R.J.); 18260068857@163.com (H.K.); gexn@cau.edu.cn (X.G.); leosj@cau.edu.cn (L.Z.); hanx0158@cau.edu.cn (J.H.); zhangyongning@cau.edu.cn (Y.Z.); yanghanchun1@cau.edu.cn (H.Y.)

**Keywords:** pseudorabies virus, Vero cell, TMT-based proteomic analysis, differentially expressed proteins

## Abstract

Suid herpesvirus 1 (SuHV-1), known as pseudorabies virus (PRV), is one of the most devastating swine pathogens in China, particularly the sudden occurrence of PRV variants in 2011. The higher pathogenicity and cross-species transmission potential of the newly emerged variants caused not only colossal economic losses, but also threatened public health. To uncover the underlying pathogenesis of PRV variants, Tandem Mass Tag (TMT)-based proteomic analysis was performed to quantitatively screen the differentially expressed cellular proteins in PRV-infected Vero cells. A total of 7072 proteins were identified and 960 proteins were significantly regulated: specifically 89 upregulated and 871 downregulated. To make it more credible, the expression of XRCC5 and XRCC6 was verified by western blot and RT-qPCR, and the results dovetailed with the proteomic data. The differentially expressed proteins were involved in various biological processes and signaling pathways, such as chaperonin-containing T-complex, NIK/NF-κB signaling pathway, DNA damage response, and negative regulation of G2/M transition of mitotic cell cycle. Taken together, our data holistically outline the interactions between PRV and host cells, and our results may shed light on the pathogenesis of PRV variants and provide clues for pseudorabies prevention.

## 1. Introduction

Pseudorabies (PR), also known as Aujeszky’s disease (AD), is one of the most notorious swine diseases and causes enormous economic losses to the pig-raising industry [1]. Typical clinical symptoms of PR include respiratory distress, nervous disorders, and reproductive failures in sows [2,3]. PR is caused by pseudorabies virus (PRV), also called suid herpesvirus 1 (SuHV-1), which belongs to the subfamily of *Alphaherpesvirus* in the family of *Herpesviridae*. The genome of PRV is about 175 kb in length and encodes over 70 viral proteins contributing to neuronal latent infection and immune modulation [4,5].

Since the first report of PR outbreak in the 1950s, PRV has spread through China over the past 70 years [6]. The intensive herd vaccination by attenuated live vaccine Bartha-K61 facilitates PR eradication, whereas strong immune pressure may accelerate the virus’s evolution and pave the way for the emergence of variants. In 2011, large scale outbreaks of PR caused by PRV variants swept China [6,7]. Subsequent studies showed that the emerging variants had higher pathogenicity, and the typical vaccine Bartha-K61 only provided limited protection against PRV variants infections [7,8]. Despite Jianle Ren et al. reporting that glycoproteins C and D of PRV variant strain HB1201 contribute individually to the escape from Bartha-K61 vaccine-induced protection [9], the pathogenesis of PRV variants remains largely unclear.

PRV has a wide host range and is capable of infecting numerous animals. Increasing evidence suggested that the newly emerged variants from 2011 were the most prevalent genotypes worldwide and most frequently involved in cross-species transmission [10]. Next-generation sequencing and regular polymerase chain reaction (PCR) confirmed the presence of PRV genomes in cerebral spinal fluid from a 43-year-old patient [11]. In addition, a patient who presented with encephalitis and pulmonary infection also tested PRV positive in his cerebrospinal fluid and vitreous humor [12]. More severely, a PRV strain was isolated from an acute human encephalitis case in 2019, confirming the interspecies transmission between pigs and humans and the replication capacity of PRV in human [13]. Other animals, including bovine and wolf, were also reported to be infected by PRV [14,15]. Understanding the interactions in-depth between PRV infection and host may provide ideas for interspecies transmission prevention.

Innate immunity is the host’s first line of defense against virus infection. When invading host cells, pathogens are recognized by specific pattern recognition receptors (PRRs) and then trigger immune responses [16]. To establish efficient infection, PRV has evolved various strategies to evade immune clearance. For example, PRV US3 degrades Bcl-2 associated transcription factor 1 to impair type I interferon production and benefit virus replication [17]; UL50 induces the degradation of type I interferon receptor via lysosomal pathway to antagonize interferon response [18]. Although NF-κB signaling pathway is activated during PRV infection, the expression of pro-inflammatory genes was inhibited [19]. Additionally, Wang et al. found that UL24 protein could abrogate tumor necrosis factor alpha (TNF-α)-mediated NF-κB activation [20]. We previously reported that PRV could dramatically enhance the dephosphorylation of eIF2α and thus promote host cell translation efficacy to facilitate its replication [21]. Higher pathogenesis and cross-species transmission ability of PRV may partly attribute to the enhanced immune evasion of PRV variants. Despite several decades of intensive study, the underlying mechanisms of PRV pathogenesis and immunomodulation still remain elusive. Hence, it is imperative to investigate the host factors involved in virus infection.

To date, proteomics is broadly applied to hunt for host factors relevant to virus infection [22]. Various animal viruses had been subjected to proteomic analysis to dissect the host factors involved in virus infection, such as porcine epidemic diarrhea virus (PEDV) [23], porcine reproductive and respiratory syndrome virus (PRRSV) [24] and porcine delta-coronavirus [25]. Tandem Mass Tag (TMT) technology, developed and launched by Thermo, is one of the most powerful quantitative methods for protein expression analysis with the highest throughput, the lowest systematic error, and the most powerful functions. In this study, TMT-based quantitative proteomics was employed to analyze protein profiles in mock- and PRV-infected Vero cells to gain insights into the virus-host interactions.

## 2. Materials and Methods

### 2.1. Cell Lines, Viruses, Chemicals, and Antibodies

African green monkey kidney cell (Vero), the immortalized porcine alveolar macrophage (CRL-2843), and porcine kidney cell (PK-15) were all cultured in Dulbecco’s modified Eagle’s medium (DMEM: Invitrogen, Carlsbad, CA, USA) containing 10% (*v/v*) fetal bovine serum (FBS, Thermo Fisher, Waltham, MA, USA) in a humidified 37 °C incubator with 5% CO_2_ and stored in our lab. PRV HB1201 (GenBank accession number: KU057086.1) was a variant strain isolated from a pig in He Bei in China. 4′, 6′-diamidino-2-phenylindole (DAPI) and TMT 16Plex were purchaseded from Thermo Fisher Scientific (Waltham, MA, USA). The primary antibodies used in this study were specific for XRCC5 (16389-1-AP, Proteintech, Rosemont, IL, USA), XRCC6 (10723-1-AP, Proteintech, Rosemont, IL, USA), β-actin (66009-1-Ig, Proteintech, Rosemont, IL, USA), VP5 (prepared in our lab), and gB (prepared in our lab). The HRP-labeled secondary antibodies against rabbit (ZB2301) and mouse (ZB2305) were all purchased from ZSGB-BIO (Beijing, China).

### 2.2. Virus Inoculation and Protein Preparation

Vero cells were grown to monolayers in 10 cm cell culture dishes and then were inoculated with PRV HB1201 at 0.1 MOI for 1 h. Sustaining culture medium DMEM containing 2% FBS was added for another 24 h. Three independent experiments were conducted as biological replicates. The protein extraction procedure is as follows: at 24 h post-inoculation (h p.i.), the medium was removed and washed with 5 mL pre-cooling PBS twice; mock- or PRV-infected Vero cells were collected using a cell scraper and piped into 1.5 mL EP tubes; protein lysate (8 M urea, 1% SDS containing protease inhibitor) was added to lyse cell membrane and sonicated for 2 min to solubilize protein further; cell lysate was used to treat protein for another 30 min on ice and centrifuged (12,000 rpm for 15 min at 4 °C) to remove cellular debris. The protein concentration was analyzed by Bradford protein assay and SDS-PAGE was performed to evaluate the overall protein quality.

### 2.3. Reductive Alkylation and TMT Labeling

Protein reductive alkylation and TMT labeling procedures were conducted according to the instructions as follows. Briefly, 100 µg protein was treated with triethylammonium bicarbonate buffer (TEAB) to the final concentration of 100 mM, and then Tris (2-carboxyethyl) phosphine (TCEP) was added to make the final concentration 10 mM for 60 min at 37 °C; 40 mM iodoacetamide was added to the final concentration and reacted in a dark room for 40 min at room temperature (RT); ice-cold acetone was added (v:v = 6:1) and reacted for 4 h at −20 °C, and the liquid was removed after centrifugation at 10,000× *g* for 20 min; sediment was dissolved with 100 µL 100 mM TEAB and digested with trypsin (m:m = 1:50) fully overnight at 37 °C; finally, TMT was added to label proteins for 2 h at RT, followed by hydroxylamine treatment for another 30 min.

### 2.4. Immunofluorescence Assay (IFA)

The IFA was performed according to the protocol mentioned previously [21]. In brief, Vero cells seeded on coverslips in a six-well plate over 90% confluence were inoculated with 0.1 MOI PRV HB1201; then, the inoculated cells were fixed with 3.7% paraformaldehyde at indicated time points for 10 min and permeabilized with 2% bovine serum albumin (BSA) containing 0.1% Triton X-100 for 10 min; 2% BSA was used to block cells for 30 min and primary monoclonal antibody specific for gB with 1:1000 dilution incubated cells for 1 h at RT and then washed with PBS three times; secondary antibodies were added at RT for 1 h in a humid chamber; after one wash, nucleus were stained with DAPI (Molecular Probes) for 10 min and washed with PBS five times for 5 min each; finally, the coverslips were observed with a Nikon A1 microscope or laser confocal microscope.

### 2.5. RNA Extraction and Real-Time PCR Analysis

Total RNAs of mock- or PRV-infected Vero cells were extracted by TRIzol reagent (Biomed, Beijing, China). The culture medium was removed and the cells in six-well plates were lysed with 750 μL TRIzol for 5 min, then 250 μL chloroform was added to separate RNA. After centrifugation at 12,000 rpm at 4 °C for 10 min, the RNA fraction was transferred into a new tube and precipitated by 0.8 volumes of isopropanol. After centrifugation for 15 min at 12,000 rpm, RNA pellets were washed twice with 75% iced ethanol and resuspended in 20 μL RNase-free H_2_O. The synthesis of cDNA was performed using Fast Quant RT Kit (With gDNase) (Tian Gen Biotech, Beijing, China) according to the manufacturer’s instructions. The cDNA samples were quantified by SYBR Green RT-qPCR Master Mix (Vazyme, Nanjing, China) and repeated three times. All reactions were carried out by the Bio-Rad PCR system. All primers used in this study are listed in Table 1. The mRNA abundance of GAPDH, XRCC5, and XRCC6 were detected by RT-qPCR assay using specific primer sets GAPDHF/GAPDHR, XRCC5F/XRCC5R, and XRCC6F/XRCC6R respectively.

### 2.6. Western Blot Analysis

PRV HB1201-infected Vero, CRL-2843, and PK-15 cells were all harvested at 24 h p.i. The cells were lysed with radioimmunoprecipitation (RIPA) lysis buffer (Beyotime, Shanghai, China) containing protease inhibitor (1 mM PMSF) for 30 min, and the supernatant was transferred to a new tube after centrifugation. The protein concentration was determined with Pierce BCA Protein Assay Kit (Thermo Fisher, Waltham, MA, USA) and separated by sodium dodecyl sulfate-polyacrylamide gel electrophoresis (SDS-PAGE). Separated protein (10 μg each channel) was transferred onto polyvinylidene difluoride (PVDF) membrane (Millipore). PVDF membranes were blocked in 5% skimmed-milk-PBST at RT for 2 h, followed by incubation with primary antibodies at 4 °C overnight. Then, the PVDF membrane were washed three times with 0.05% PBST for 5 min each at a rotator and incubated with the HRP-conjugated secondary antibodies at 1:3000 dilution. After three washes, the membranes were incubated with ECL chemiluminescence detection kit (Pierce) for 2 min, and finally exposed to a chemiluminescence apparatus (Bio-Rad, Hercules, CA, USA).

### 2.7. Virus Titration

Viruses were serially diluted 10-fold with DMEM containing 2% FBS and inoculated into Vero cells at 90% confluence in 96-well culture plates. 72 h p.i. or later, the virus titers were calculated based on the cytopathic effects (CPE) according to the Reed-Muench method. Virus titers were determined from at least three independent experiments.

### 2.8. Data Analysis

All data were processed with GraphPad Prism 6 (GraphPad Software Inc., San Diego, CA, USA). The student’s *t*-test or non-parametric test was used to analyze the difference between the values of two groups. A value of *p* < 0.05 was considered statistically significant.

## 3. Results

### 3.1. Kinetics of PRV HB1201 Replication in Vero Cells

**Figure 1:**
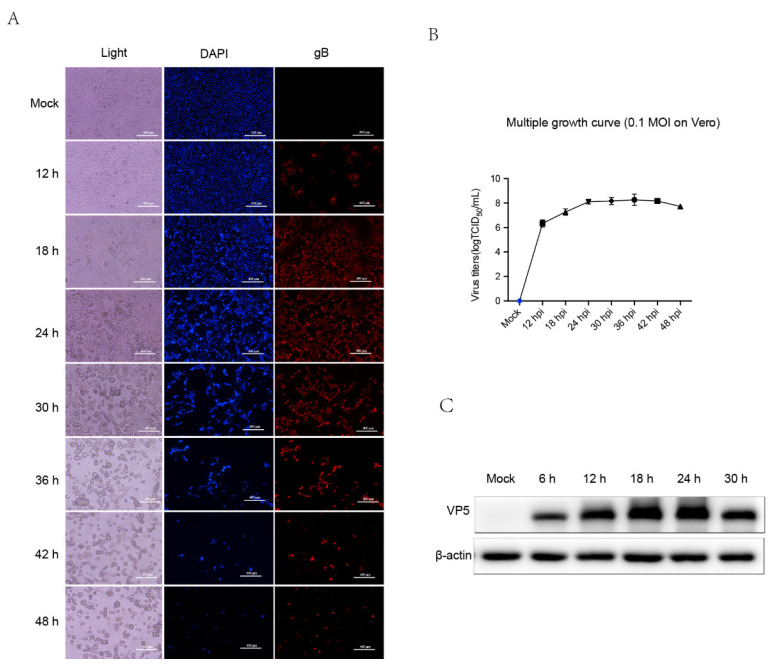
The replication of PRV variant HB1201 in Vero cells. (**A**) PRV at 0.1 MOI infects Vero cells and CPE was observed in microscopy at various time points. Meanwhile, IFA was also applied to view the efficiency of virus infection with antibody against gB protein. (**B**) The whole cells infected with 0.1 MOI virus were collected at indicated time points and tittered by TCID_50_. (**C**) Vero cells infected with 0.1 MOI PRV were collected and the whole cell lysis was subjected to western blot analysis to detect the expression of VP5.

Efficient viral infection and relatively mild cell collapse are critical factors for optimal sampling. PRV HB1201 could cause severe CPE and subsequently cell collapse on Vero cells, thus relative lower MOI (MOI = 0.1) was applied to infect Vero cells. To screen the optimal time points of sampling, the kinetics of PRV replication in Vero cells were determined at various time points by TCID_50_. As shown in Figure 1B, the virus titers were up to 10^8^ TCID_50_/mL at 24 h p.i., similar to that at 30 to 48 h p.i., indicating PRV could propagate in Vero cells efficiently, and the virus titers reached a plateau at 24 h p.i. (Figure 1B). Furthermore, IFA results showed that gB positive cells increased as the infection progressed. Notably, most cells were infected at 24 h p.i., and the gB positive cells decreased after 30 h p.i. due to excessive cell collapse (Figure 1A). Meanwhile, the CPE was observed microscopically at various time points. Compared with mock-infected cells, PRV-Infected cells developed slightly visible CPE at 12 h p.i. and CPE were fairly apparent at 24 h p.i. (Figure 1A). Cell collapse soars from 30 h p.i., and many of the cells were detached and floated in the medium. In addition, the expression of viral capsid protein VP5 was detected by western blot. The level of VP5 increased gradually as infection progressed (Figure 1C). However, VP5 expression level decreased slightly at 30 h p.i. compared to that at 24 and 18 h p.i. This may result from cell detachment and virus release into the medium (Figure 1A). Based on the results above, Vero cells infected with 0.1 MOI PRV for 24 h p.i. were regarded as optimal sampling time points and subjected to the following proteomic analysis.

### 3.2. Protein Profiles Determined by TMT/MS Analysis

**Figure 2:**
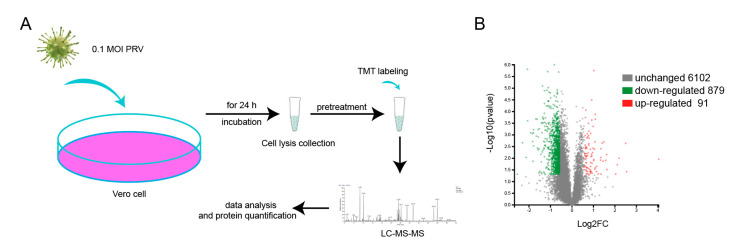
Overview of proteomic analysis procedure and DEPs. (**A**) The workflow of proteomic analysis. Vero cells were infected with 0.1 MOI PRV for 24 h and the whole cells lysis was collected after centrifugation at 12,000 rpm for 5 min. After reductive alkylation, the protein was labeled with TMT. Finally, the samples were subjected to liquid chromatography tandem mass spectrometry and bioinformatics analysis. (**B**) A total of 7022 proteins were identified, among which 89 proteins were markedly upregulated (red dots), 879 proteins were downregulated (green dots), and the remaining 6102 proteins stayed constant (gray dots). Proteins were considered significantly differently expressed when *p* value was less than 0.05 and fold change was less than 0.67 or more than 1.5 in this study.

Proteomics is a systematic approach to study the virus-host interactions. To identify the differentially expressed proteins (DEPs) between mock- and PRV-infected cells, TMT-based quantitative proteomic analysis was performed, and the workflow is shown as Figure 2A. A total of 7072 cellular proteins were identified and quantified at 24 h p.i., among which 91 proteins were significantly upregulated and 879 proteins were downregulated compared to those in mock-infected Vero cells (Figure 2B) according to the criteria (*p*-value < 0.05 and fold change >1.5 or fold change <0.67). In addition, the top 20 upregulated and top 20 downregulated proteins are listed in Table 2 and Table 3, respectively. Three technical replicates were carried out to improve the reliability of our data.

### 3.3. Validation of TMT/MS Data by Western Blot and RT-qPCR

**Figure 3:**
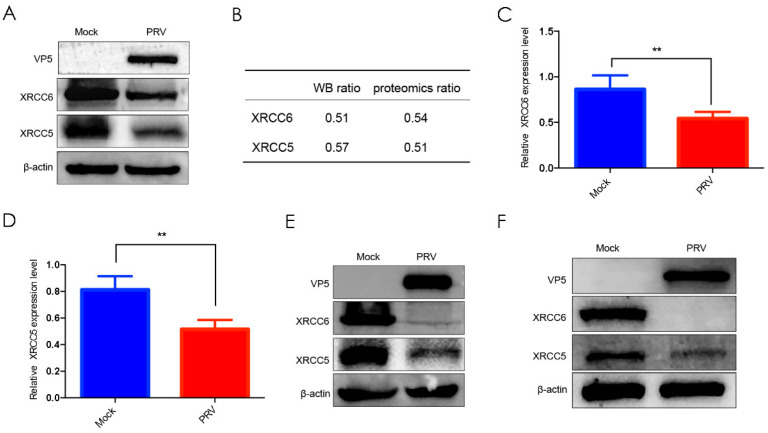
Validation of proteomics data by western blot and RT-qPCR. (**A**) Vero cells infected with PRV for 24 h were collected and western blot was performed to detect the expression of XRCC5 and XRCC6 with corresponding antibodies. (**B**) The western blot and proteomics ratio of XRCC5 and XRCC6. (**C**) Relative XRCC6 transcription in Vero cells. (**D**) Relative XRCC5 transcription in Vero cells. (**E**) The expression of XRCC5 and XRCC6 in PK-15 infected with PRV. (**F**) The expression of XRCC5 and XRCC6 in CRL-2843 infected with PRV. ** indicates significance at a 99% confidence interval (*p* < 0.01).

To verify TMT/MS data, X-ray repair cross-complementing protein 5 (XRCC5) and X-ray repair cross-complementing protein 6 (XRCC6) were analyzed by western blot in both mock- and PRV-infected Vero cells. The two proteins were selected for validation for the following reasons: they were downregulated significantly; they were closely related and involved in DNA repair process, which was a general cellular response during herpes virus infection [26]; and antibodies against them were commercially available. Western blot results showed that the protein level of XRCC5 and XRCC6 both decreased in PRV-infected Vero cells (Figure 3A). Then, Image J software was applied to quantify protein levels, and the ratios of the XRCC5 and XRCC6 between mock-and PRV-infected Vero cells coincided with proteomic data (Figure 3B). Moreover, the levels of XRCC5 and XRCC6 in PK-15 and CRL-2843 cells were also reduced (Figure 3E,F), indicating PRV-mediated XRCC5 and XRCC6 reduction was in a cell type-independent manner.

In addition, the transcription level of XRCC5 and XRCC6 were also analyzed by RT-qPCR. Consistently, the mRNA level of XRCC5 and XRCC6 markedly decreased in virus-infected Vero cells compared with that in mock-infected cells (Figure 3C,D), suggesting that PRV-mediated XRCC5 and XRCC6 downregulation might result from transcription inhibition.

### 3.4. GO Analysis of The DEPs

GO annotation analysis could classify the tested proteins in three aspects: biological process (BP), cellular component (CC), and molecular function (MF). To dissect the function of DEPs, GO functional analysis revealed that 89 upregulated proteins and 871 downregulated proteins were involved in 12 biological processes (Figure 3A), including cellular processes, single-organism processes, metabolic processes, biological processes, regulation of biological processes, and so on; within the CC category, the DEPs were well distributed in different cell components, including cell parts, cells, organelle, organelle parts, and so on; in the MF category, the DEPs were involved in binding function.

**Figure 4:**
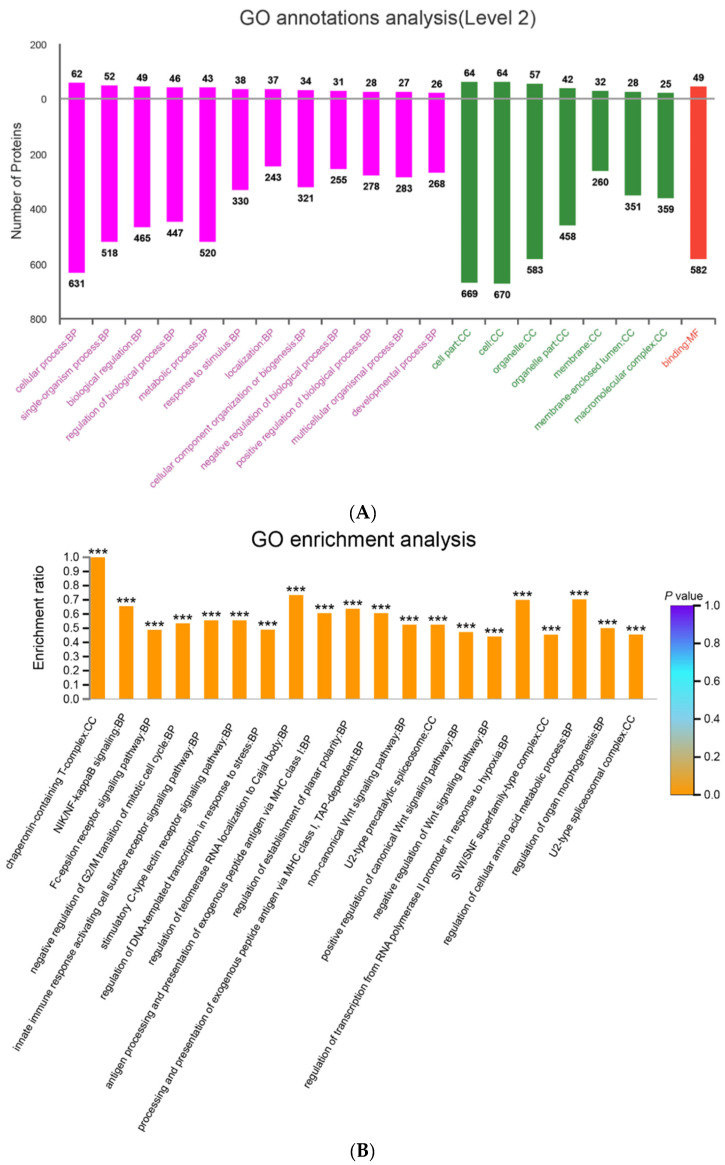
GO annotation and GO enrichment analysis of DEPs between mock- and PRV-infected Vero cells. (**A**) Up- and downregulated proteins are classified into three categories, respectively, by GO analysis: biological process (BP), cellular component (CC), and molecular function (MF). The x-axis represents the specific categories in BP, CC, and MF. The numbers on the y-axis indicate proteins in the category. (**B**) DEPs were subjected to GO enrichment analysis and the top 20 GO terms are listed on the x-axis. The y-axis indicates the enrichment ratio of DEPs and different colors represent different *p* values. *** indicates significance at a 99.9% confidence interval (*p* < 0.001).

In addition, GO enrichment analysis demonstrated that DEPs were mostly enriched in chaperonin-containing T-complex within the CC category. Furthermore, the majority of DEPs were enriched in the BP category, such as NIK/NF-κB signaling, Fc-epsilon receptor signaling pathway, negative regulation of G2/M transition of mitotic cell cycle, and innate immune response activating cell surface receptor signaling pathway (Figure 4B).

### 3.5. KEGG Functional Annotation of DEPs

**Figure 5:**
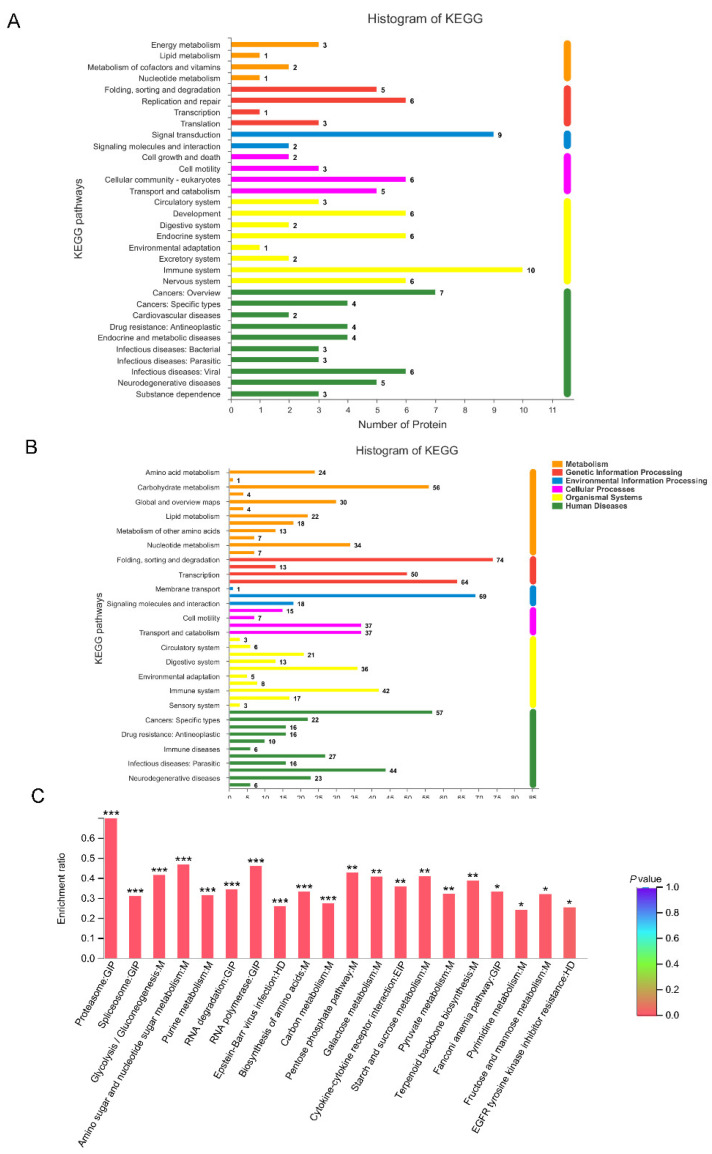
(**A**) The 89 upregulated proteins are classified into six main categories by KEGG analysis: metabolism, genetic information processing, environmental information processing, cellular processes, organismal systems, and human diseases. The x-axis indicates the numbers of proteins within particular categories. The y-axis indicates the specific pathways within six main categories. (**B**) The 871 downregulated proteins are classified in the same manner as Figure A: (**C**) KEGG pathway enrichment analysis of DEPs. The x-axis indicates the name of the KEGG pathways, the y-axis indicates the enrichment ratio (there were no apparent differences between 0.01 and 0.05, so the colors look similar and can’t be distinguished by the naked eye). * indicates significance at a 95% confidence interval (*p* < 0.05), ** indicates significance at a 99% confidence interval (*p* < 0.01), *** indicates significance at a 99.9% confidence interval (*p* < 0.001).

KEGG pathway analysis was performed to further explore the underlying signaling pathways or functions among DEPs. As shown in Figure 5A, the 89 upregulated proteins participated in 32 pathways, and the top three were related to the immune system, signal transduction, and cancer. Meanwhile, the 871 downregulated proteins were involved in 44 pathways, and the top three were the “folding, sorting, and degradation of protein”, signal transduction, and translation (Figure 5B).

KEGG enrichment analysis were also conducted to analyze the enriched signaling pathways in DEPs. Among all 970 DEPs, 20 pathways were significantly enriched, and the top three were proteasome, amino sugar and nucleotide sugar metabolism, and RNA polymerase (Figure 5C).

### 3.6. COG Annotation of DEPs

**Figure 6:**
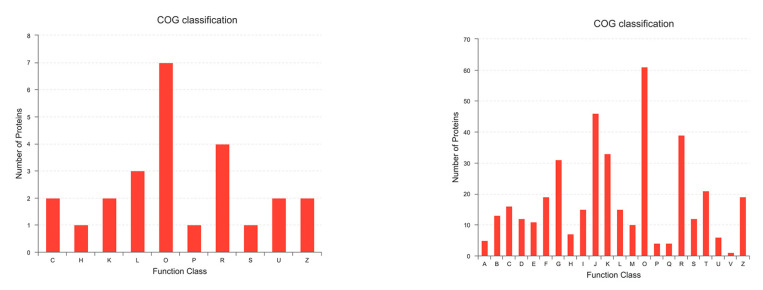
The COG annotation of significantly upregulated proteins (**left panel**) and downregulated proteins (**right panel**). The x-axis indicates the COG functional classification (presented with A to Z): (A) RNA processing and modification; (B) Chromatin structure and dynamics; (C) Energy production and conversion; (D) Cell cycle control, cell division, and chromosome partitioning; (E) Amino acid transport and metabolism; (F) Nucleotide transport and metabolism; (G) Carbohydrate transport and metabolism; (H) Coenzyme transport and metabolism; (I) Lipid transport and metabolism; (J) Translation, ribosomal structure, and biogenesis; (K) Transcription; (L) Replication, recombination, and repair; (M) Cell wall/membrane/envelope biogenesis; (O) Posttranslational modification, protein turnover, and chaperones; (P) Inorganic ion transport and metabolism; (Q) Secondary metabolites biosynthesis, transport, and catabolism; (R) General function prediction only; (S) Function unknown; (T) Signal transduction mechanisms; (U) Intracellular trafficking, secretion, and vesicular transport; (V) Defense mechanisms; (Z) Cytoskeleton. The y-axis indicates the protein number of particular functional classifications.

The COG database is able to predicate the function of proteins based on protein sequence. To categorize the functions of DEPs, COG analysis was performed. As shown in Figure 6 (left panel) 10 categories were involved in upregulated proteins. In particular, seven proteins were related to posttranslational modification, protein turnover, and chaperones; four proteins were classified into general function prediction only; three proteins were related to replication, recombination, and repair; two proteins were relevant to energy production and conversion, transcription, intracellular trafficking, secretion, vesicular transport, and so on. In addition, 22 categories were involved in 879 downregulated proteins: 61 proteins were related to posttranslational modification, protein turnover, and chaperones; 46 proteins were relevant to translation, ribosomal structure, and biogenesis; 39 proteins were classified into general function prediction only shown in Figure 6 (right panel). Further research is imperative to characterize the involvement of these categories during PRV infection.

## 4. Discussion

PRV variant HB1201 exhibits higher pathogenicity, and its pathogenesis remains poorly defined. Nowadays, proteomics has been broadly used in profiling cellular protein expression patterns in virus-infected cells. In this paper, a TMT-based quantitative proteomics approach was applied, and we revealed striking protein profile shifts in PRV-infected Vero cells compared with those in mock-infected cells.

In the present study, a total of 7072 proteins were identified in whole Vero cells, among which 980 proteins were differentially expressed at 24 h p.i. Among the top 20 upregulated proteins, non-homologous end joining-1 (NHEJ-1) markedly induced an over five-fold change (Table 2), which is reported to be involved in DNA repair [27]. DNA viruses replicates their genomes in the nuclei of cells, and the mass accumulation of viral DNA genome in the nucleus may trigger host cell DNA damage responses. For example, intensive studies showed that herpes virus may engage components of DNA damage response to enhance its replication, while some of the DNA repair components are antiviral [28,29,30]. In our analysis, the expression of two DNA repair-related proteins, XRCC5 (Ku80) and XRCC6 (Ku70), were both shown to be reduced in PRV-infected cells. Moreover, western blot and RT-qPCR results supported the proteomic data at both protein and transcription level. XRCC5 and XRCC6 comprise the heterodimer, which recognizes and binds to double strand DNA break ends, and then promotes non-homologous end joining [30] or induces innate immune defenses against DNA virus infection [31,32]. Previous reports showed that XRCC6 not only modulated human T lymphotropic virus type 1 (HTLV-1) replication [33], but also regulated DNA virus-mediated innate immune response [34]. However, the expression of XRCC6 was significantly upregulated in HTLV-1-infected cells compared with PRV. We hold that PRV, with its larger genome, encodes more proteins and evolves more sophisticated strategies to evade host immune clearance by targeting XRCC5 and XRCC6. As compensation, many other tricks have been developed instead by HTLV-1. For example, HTLV-1 Tax could impair K63-linked ubiquitination of STING to evade host innate immunity [35]; HTLV-1 Tax blocks IRF3 phosphorylation through the interaction with and inhibition of TBK1 kinase [36]. These results above indicated that the DNA damage repair signaling pathway might be closely related to virus infection.

Our previous study showed that PRV infection induced the phosphorylation of PERK; however, the expression of GRP78 stayed unaltered [21], indicating that other host factors might alleviate the intensity of unfolded protein responses (UPR). The endoplasmic reticulum (ER) is a major factor of glycoprotein synthesis, and the excessive expression of glycoprotein may activate UPR [37]. According to proteomic data, the expression of seven-transmembrane superfamily member 3 (TM7SF3), engaged in the attenuation of cellular stress and the subsequent UPR [38], was significantly induced. TM7SF3 is a downstream target of p53 [38], which is involved in innate immune response regulation, cell cycling, DNA repair, and apoptosis [39,40]. Although Xun Li et al. reported that overexpression of p53 positively regulated PRV replication both in vivo and in vitro [41], many questions were still hanging in the air: for example, the expression level and activation status of p53 during PRV infection and its contributions to TM7SF3 overexpression. Most importantly, the biological significance of TM7SF3 on UPR and virus replication were imperative to be elucidated.

Innate immune response, particularly type I interferon production and inflammatory cytokines secretion, is the first line to fight against pathogen invasion. PRRs recognize pathogen-associated molecular patterns (PAMPs) and then trigger innate immune responses. PRV is a common pathogen in multiple animal species and has even been isolated from human patients [42], thus attention should also be paid to the protein profile shifts in Vero lines. Vero cells are type I interferon-deficient, so inflammatory responses are emphasized in this paper. Our results showed that the NIK/NF-κB signaling pathway was markedly enriched by GO enrichment analysis. It was reported that the virulent PRV variant induced substantial lethal inflammatory response by TRL2, while the attenuated live vaccine of PRV lost the ability to activate an inflammatory response [13,43]. The abnormal inflammatory responses mediated by PRV variants might contribute to its pathogenicity. KEGG enrichment analysis showed that DEPs were significantly enriched in proteasome (Figure 5C); in particular, 28 DEPs were relevant to proteasome. Proteasome was reported to shape innate immune response and regulate the production of inflammatory cytokines [44]. Therefore, we proposed that PRV could modulate inflammatory response via regulating 26s proteasome non-ATPase regulatory subunits and other proteasome-related proteins expression. During PRV propagation, there are amounts of viral proteins synthesized in cells. We hold that the proteasome-related proteins may also be involved in useless or damaged protein degradation to maintain cellular homeostasis. In addition, cells may recruit proteasome to degrade viral proteins by ubiquitinating them and achieve an antiviral during PRV infection. Furthermore, the PRRs regulator tripartite motif-containing protein 40 (TRIM40) was also significantly down-regulated, indicating PRV might subvert innate immune responses by inhibiting PRRs activation. Additionally, the enzymatic activity of cGAS is tightly regulated by XRCC5 and XRCC6 to maintain immune homeostasis [45,46]. PRV is enveloped, and multiple processes all require lipids, such as virus-cell membrane fusion and virus budding. Our results showed that the elongation of very long chain fatty acids protein 1 (ELOVL1), involved in unsaturated fatty acid biosynthetic process [47], was significantly upregulated during PRV infection. This suggests that cellular lipids metabolism may take part in PRV propagation and pathogenesis. In addition, E3 ubiquitin-protein ligase NEDD4 was also significantly upregulated in PRV-infected cells. NEDD4 is essential for neural development and homeostasis of neural circuit excitability during neuronal ER stress [48], indicating this may be a protective mechanism to maintain cell homeostasis and normal biological functions during PRV infection. Taken together, the interactions between PRV infection and innate immune responses are complex and need further investigation.

This study systematically analyzed the protein profiles of PRV-infected Vero cells using a TMT-based proteomic analysis method. Eighty-nine upregulated and 871 downregulated proteins were identified, and biological analysis demonstrated that various cellular processes were involved in PRV-infected cells, including cellular processes, single-organism processes, metabolic processes, biological processes, regulation of biological processes, and so on. Unfortunately, our analysis of DEPs remains only instructional, and the elucidation of their biological functions is required. This research will help to deepen the understanding of the virus pathogenesis and host immune responses.

## Figures and Tables

**Table 1:** Primers used in this study.

Primer Name	Primer Sequence
XRCC6-F	GCTCCTTGGTGGATGAGTTT
XRCC6-R	CTTGCTGATGTGGGTCTTCA
XRCC5-F	TGACTTCCTGGATGCACTAATCGT
XRCC5-R	TTGGAGCCAATGGTCAGTCG
GAPDH-F	CCTTCCGTGTCCCTACTGCCAAC
GAPDH-R	GACGCCTGCTTCACCACCTTCT

**Table 2:** Top 20 up-regulated proteins.

Accession	Description	FC (P_24h/M_24h)	*p* Value (P_24h/M_24h)	Significant
XP_007997295.1	4-hydroxybenzoate polyprenyltransferase, mitochondrial	16.440879	0.0111	Yes
XP_008000412.1	ATP synthase subunit gamma, mitochondrial isoform X2	5.82243	0.03774	Yes
XP_007964526.1	non-homologous end-joining factor 1	5.638689	0.002289	Yes
XP_007966188.1	transmembrane 7 superfamily member 3 isoform X1	4.570457	0.009848	Yes
XP_007985885.1	DNA-directed RNA polymerase III subunit RPC5 isoform X1	4.318305	0.01897	Yes
XP_008001057.1	proton myo-inositol cotransporter	4.10077	0.04733	Yes
XP_007978034.1	hemoglobin subunit alpha	3.092117	0.03053	Yes
XP_007959284.1	bromodomain-containing protein 9 isoform X1	3.054434	0.006955	Yes
XP_008010294.1	testis-expressed sequence 2 protein isoform X1	2.974047	0.01225	Yes
XP_007995408.1	relA-associated inhibitor isoform X1	2.802074	0.00199	Yes
XP_008014790.1	tropomodulin-2 isoform X1	2.732538	0.01336	Yes
XP_007965594.1	myeloid leukemia factor 2	2.647029	0.002251	Yes
XP_007958764.1	kinesin-like protein KIF16B isoform X1	2.550698	0.003898	Yes
XP_007958522.1	conserved oligomeric Golgi complex subunit 3	2.549788	0.04741	Yes
XP_007997053.1	serum albumin	2.544889	0.009266	Yes
XP_007977282.1	elongation of very long chain fatty acids protein 1	2.521127	0.01201	Yes
XP_008008965.1	vitronectin	2.339382	0.04719	Yes
XP_008012665.1	calcium signal-modulating cyclophilin ligand	2.218158	0.02212	Yes
XP_007995769.1	splicing factor, arginine/serine-rich 19 isoform X1	2.208965	0.037	Yes
XP_008014703.1	E3 ubiquitin-protein ligase NEDD4 isoform X3	2.12792	0.000212	Yes

**Table 3:** Top 20 down-regulated proteins.

Accession	Description	FC (P_24h/M_24h)	*p* Value (P_24h/M_24h)	Significant
XP_007995562.1	glioma tumor suppressor candidate region gene 2 protein	0.156304	0.007612	Yes
XP_008007884.1	UAP56-interacting factor isoform X1	0.171554	0.04785	Yes
XP_007975472.1	thioredoxin-interacting protein	0.202567	0.002175	Yes
XP_007971461.1	tripartite motif-containing protein 40	0.2282	0.009207	Yes
XP_008016505.1	general transcription factor II-I	0.237577	0.000002	Yes
XP_008016152.1	wolframin	0.239067	0.000248	Yes
XP_007972702.1	structural maintenance of chromosomes flexible hinge domain-containing protein 1 isoform X1	0.263824	0.000806	Yes
XP_007980315.1	epsilon-sarcoglycan isoform X1	0.280037	0.000878	Yes
XP_007966031.1	zinc finger protein AEBP2 isoform X1	0.280576	0.001929	Yes
XP_008013602.1	histone-lysine N-methyltransferase, H3 lysine-36 and H4 lysine-20 specific isoform X1	0.284503	0.01345	Yes
XP_007962440.1	regulator of G-protein signaling 10 isoform X1	0.287038	0.000822	Yes
XP_007982422.1	vasorin	0.289883	0.000601	Yes
XP_007977511.1	probable U3 small nucleolar RNA-associated protein 11 isoform X1	0.289894	0.002949	Yes
XP_008001366.1	bax inhibitor 1	0.311135	0.000205	Yes
XP_007962616.1	antigen KI-67 isoform X1	0.316703	0.001139	Yes
XP_007960624.1	ribosome biogenesis protein BMS1 homolog	0.323469	0.000212	Yes
XP_008016787.1	zinc finger and SCAN domain-containing protein 21 isoform X2	0.328314	0.003606	Yes
XP_008012144.1	alpha-protein kinase 2 isoform X1	0.332672	0.00059	Yes
XP_008013174.1	treacle protein isoform X1	0.334286	0.004324	Yes
XP_008007268.1	solute carrier organic anion transporter family member 2A1	0.338268	0.001218	Yes

## Data Availability

The proteomic data is available with the link: https://pan.baidu.com/s/12D_7pNG1VP79E_aNfgi-LQ (Password: 39ht, accessed on 1 March 2022).
